# Comparative Study of Phytochemical, Antioxidant, and Cytotoxic Activities and Phenolic Content of *Syzygium aqueum* (Burm. f. Alston f.) Extracts Growing in West Sumatera Indonesia

**DOI:** 10.1155/2021/5537597

**Published:** 2021-06-14

**Authors:** Afrizal Itam, Mutia Siska Wati, Vina Agustin, Nursal Sabri, Rafika Aris Jumanah, Mai Efdi

**Affiliations:** Department of Chemistry, Faculty of Mathematic and Natural Sciences, Andalas University, Padang, Indonesia

## Abstract

*Syzygium aqueum*, consisting of various fruit colors, is one of the plants that have been used as traditional medicine. This study aims to evaluate and compare phytochemical, antioxidant, and cytotoxic activities and total phenolic content of leaves and stem bark extracts of *S. aqueum* with pink and red fruits, in order to identify the best extract that can be used as a natural antioxidant. Phytochemical constituents were evaluated qualitatively using chemicals, while cytotoxic activities were identified using the brine shrimp lethality test. Total phenolic content was determined via the Folin–Ciocalteu method. Leaves and stem bark of *S. aqueum* contained flavonoids, phenolics, and triterpenoids, but the stem bark also contained saponins and alkaloids. Methanol and ethyl acetate extracts of leaves and stem bark were categorized as very powerful antioxidants to DPPH (IC_50_ 9.71–38.69 *μ*g/mL) and hydrogen peroxide (IC_50_ 16.44–44.02 *μ*g/mL), while hexane extracts were inactive. Methanol, ethyl acetate, and hexane extracts of leaves and stem bark were categorized as moderately cytotoxic to *A. salina* larvae (LC_50_ 104.04–440.65 *μ*g/mL). Comparing leaves and stem barks, antioxidant and cytotoxic activities of stem bark extracts were higher than those of leaves extracts. Total phenolic content of leaves extracts was higher than that of stem bark extracts where the order of total phenolic content progressed from methanol extracts > ethyl acetate extracts > hexane extracts. Therefore, the stem bark of *S. aqueum* was identified as the better source of natural antioxidant compared with the leaves.

## 1. Introduction

*Syzygium aqueum* (family: *Myrtaceae*) is a plant in which its fruits are favored by many. It is easy to grow and is usually planted in house yards not only for its fruits but also for protection purposes. Being native to Indonesia and Malaysia, the fruit of this plant is mainly known as the water jamboo, but other common names include water apple, bell fruit, water cherry, or watery rose apple. There are different variations of this plant with the main difference being the shape and color of the fruits. Various parts of *S. aqueum* have been utilized in traditional medicine due to the antibiotic activities that are present [1, 2]. Palanisamy et al. [3] reported that ethanolic extracts of *S. aqueum* show tyrosinase inhibitory activities. Other researchers reported six isolated flavonoid compounds from the ethanolic extracts of this plant, namely 4-hydroxybenzaldehyde, myricetin-3-O-rhamnoside, europetin-3-O-rhamnoside, phloretin, myrigalone-G, and myrigalone-B [2, 4] with two of these flavonoids (i.e., myricetin-3-O-rhamnoside and europetin-3-O-rhamnoside) showing high inhibitory activities as antihyperglycemic agents. This plant also contains terpenoids, tannins, and saponins [2].

Plants used as traditional medicines are called herbs. Scientific literature suggests that 80% of Asian and African (or 80% of the world's population) use traditional medicine to meet their primary healthcare needs [5]. Phytonutrient or phytochemistry originating from the plants results in various activities that benefit the health of humans and the plants themselves. These compounds are known as secondary metabolites such as alkaloids, terpenoids, steroids, and flavonoids. Among them, flavonoids, sometimes called phenolic compounds, exhibit antioxidant properties, which allow them to produce anti-inflammation, antiallergenic, antivirus, antiaging, and anticarcinogenic effects [6, 7].

Two groups of antioxidants (i.e., natural antioxidants and synthetic antioxidants) are often employed in medicine and food especially those containing fats and oils to prevent oxidation. Prominent synthetic antioxidants are butylated hydroxyanisole (BHA) and butylated hydroxytoluene (BHT), which have been extensively used in the food, cosmetic, and therapeutic industry. However, these synthetic antioxidants are carcinogenic, highly volatile, and instable at high temperature, potentially causing various fatal diseases. Therefore, a natural antioxidant is required as an alternative that is appropriate for dietary intake, shows no hazardous effects to the human body, and is obtainable at a low cost [8].

Considering this, *S. aqueum* is viewed as a great potential in traditional medicine as a source of natural antioxidants. While there are many variations of *S. aqueum*, each different in shape and color of the fruit, there is no study reported on the *S. aqueum* stem bark. Hence, the purpose of this study is to evaluate and compare the phytochemical, antioxidant, and cytotoxic activities as well as the total phenolic content of the leaves and stem bark of *S. aqueum* with pink and red fruits. Leaves and stem barks were extracted using methanol, ethyl acetate, and hexane solvent to obtain extracts containing components with different polarity (i.e., polar, semipolar, and nonpolar). Methanol, ethyl acetate, and hexane extracts are categorized as polar, semipolar, and nonpolar, respectively.

## 2. Materials and Methods

### 2.1. Materials and Chemicals

The filtrate obtained from maceration was evaporated using the rotary evaporator Heidolph Basis Hei-VAP HL (Germany), while antioxidant activities were determined using the spectrometer UV/VIS Shimadzu Pharma Spec UV-1700 Series. The medium used for brine shrimp growth was prepared using a small tank consisting of two compartments filled with sea water. Hexane, ethyl acetate, and methanol solvents for extraction were obtained via distillation. Iron (III) chloride, hydrogen chloride, sulfuric acid, sodium hydroxide, ammonia, magnesium powder, acetic anhydride, chloroform, Mayer's reagent, sodium carbonate, and methanol p.a. were purchased from Merck KGaA (Darmstadt, Germany). Gallic acid, Folin–Ciocalteu, and 2,2-diphenyl-1-picrylhydrazyl were purchased from Sigma Chemical Company (St. Louis MO, USA).

### 2.2. Preparation of Samples

Leaves and stem bark of *S. aqueum* with pink and red fruits were collected from West Sumatera, Indonesia (1° 33′ 22″ S, 100° 14′ 4″ E). These samples were identified, and the specimens were stored in the Herbarium of Biology Department, Andalas University. These samples were cleaned, dried at room temperature, and subsequently grinded into powder.

### 2.3. Phytochemical Screening

Phytochemical screening of the leaves and stem bark of *S. aqueum* were done based on the methods of Itam et al. [9] and Gul et al. [10] with adjustment. 5 g of powdered leaves and stem bark samples were macerated with 50 mL of methanol in a separated flask for 30 minutes at boiling point. The mixtures were filtered, and the filtrates were concentrated. 5 mL each of chloroform and water were added to the residues, which were then shaken. After leaving the mixtures for several minutes, two layers appeared. Phenolics, flavonoids, and saponins were identified using the upper layer (aqueous layer), while steroids and triterpenoids were identified using the lower layer (chloroform layer).

#### 2.3.1. Phenolics Test

1 mL of the aqueous layer was transferred into a test tube using a pipette, with 3 drops of 1% iron (III) chloride solution added to this mixture. The presence of phenolics compounds was indicated with a blue or green solution.

#### 2.3.2. Flavonoids Test

1 mL of the aqueous layer was transferred into a test tube using a pipette followed by a few milligram of magnesium powder and 1-2 drops of concentrated hydrogen chloride. The presence of flavonoids was indicated by a red solution.

#### 2.3.3. Saponins Test

2 mL of the aqueous layer was transferred using a pipette into a test tube that was then shaken for several minutes. The presence of saponins was indicated by the formation of frothing that showed no loss after 2-3 drops of hydrogen chloride were added.

#### 2.3.4. Steroids and Triterpenoids Test

1 mL of the chloroform layer was transferred into a test tube using a pipette followed by the addition of 3 drops of acetic anhydride. Three drops of concentrated sulfuric acid were added along the wall of the test tube. The presence of steroid was indicated by the appearance of a bluish-violet ring, while the presence of triterpenoid was indicated by a reddish-brown appearance.

#### 2.3.5. Coumarins Test

About 1 g of powdered leaves and stem bark samples were macerated using 10 mL of methanol in a separated flask at boiling point for 5 minutes and were then filtered. The filtrates were thin-layer chromatographed using ethyl acetate as the mobile phase. The presence of coumarins was indicated by the appearance of a blue fluorescence under ultraviolet light at 254 and 356 nm. After being sprayed with 1% sodium hydroxide solution, the intensity of fluorescence would increase.

#### 2.3.6. Alkaloids Test

About 1 g of powdered leaves and stem bark samples were macerated using 10 mL of 0.05 M chloroform-ammonia in a separated flask for 10 minutes and were then filtered. 2 mL of these filtrates were added with 2 mL of 2 N sulfuric acid. After being shaken, these mixtures were left for a few minutes to form two layers. 1 mL of the upper layer (acidic layer) was pipetted into another test tube, with Mayer's reagent added to this mixture. The presence of alkaloids was indicated by the formation of white precipitate.

### 2.4. Extraction of Leaves and Stem Bark of *S. aqueum*

The method of extraction of *S. aqueum* leaves and stem barks was adopted from Dissanayake et al. [11] with modification. 200 g of every sample (powdered leaves and stem bark of *S. aqueum*) was macerated with 200 mL of hexane, ethyl acetate, and methanol at room temperature in separate flasks overnight. The extracts were filtered and subsequently concentrated under reduced pressure using a rotary evaporator at 50°C. The concentrated extracts were weighed, and the percentages of extracts were calculated. These concentrated extracts were stored in a tight container and kept in a freezer.

### 2.5. Determination of Antioxidant Activities Using DPPH Free Radical Method

Antioxidant activities determined using DPPH free radicals were based on the methods of Itam et al. [9] and Ortega-Vidal et al. [12] with a few modifications. 10 mg of hexane, ethyl acetate, and methanol leaves and stem bark extracts were dissolved with a total methanol volume of 10 mL in separate volumetric flasks (concentration of 1,000 *μ*g/mL). These extracts were made in various concentrations: 5, 10, 20, 30, 40, and 50 *μ*g/mL for methanol and ethyl acetate extracts and 300, 400, 500, 600, 700, and 800 *μ*g/mL for hexane extracts in methanol solvent. Then, 3 mL solution of DPPH in 0.1 mM methanol was mixed with 2 mL of each extract solution in different tubes. These mixtures were left for 30 minutes in the dark at room temperature, and the absorbance was measured using the spectrophotometer UV-VIS 1700 Series at 517 nm. 3 mL of 0.1 mM DPPH and 2 mL of methanol were used as controls, while ascorbic acid was used as the positive control. The following equation was used to calculate the free radical scavenging activity (FRSA, %) of the extracts:

(1) $$\begin{array}{c} \text{FRSA}\left(\text{\%}\right)=\left[\frac{\left(A_{\text{control}}-A_{\text{sample}}\right)}{A_{\text{control}}}\right]\times 100\text{,} \end{array}$$

where *A*_sample_ is the absorbance of sample solution and *A*_control_ is the absorbance of the control solution. The equation of regression from the calibration curve obtained via the extrapolation of extract concentrations (*X*-axis) versus the percentage of radical scavenging activities (*Y*-axis) was used to calculate the inhibition concentration 50% (IC_50_).

### 2.6. Determination of Antioxidant Activities Using Hydrogen Peroxide Method

The scavenging ability of extracts to hydrogen peroxide was determined based on the methods of Sen et al. [13] and Ekin et al. [14] with slight modifications. 100 *μ*g/mL hexane, ethyl acetate, and methanol extract solutions were prepared in methanol as stock solutions, and a 40 mM hydrogen peroxide solution in phosphate buffer (pH 7) was prepared. Various concentrations of leaves and stem bark extracts, ranging from 5 *μ*g/mL to 80 *μ*g/mL, were prepared from these stock solutions. 2 mL of each extract solution was pipetted into 3.4 mL of phosphate buffer (pH 7) solution in different tubes. Then, 0.6 mL of 40 mM hydrogen peroxide was pipetted into every mixture. These mixtures were incubated for 10 minutes, and the absorbance was measured at 230 nm using the spectrometer UV/VIS against phosphate buffer pH 7 as blank. The control used was 2 mL of methanol, 3.4 mL of phosphate buffer (pH 7) solution, and 0.6 mL of 40 mM hydrogen peroxide. The hydrogen peroxide scavenging activity (HPSA, %) of extracts was calculated using the following equation:

(2) $$\begin{array}{c} \text{HPSA}\left(\text{\%}\right)=\left[\frac{\left(A_{\text{control}}-A_{\text{sample}}\right)}{A_{\text{control}}}\right]\times 100\text{,} \end{array}$$

where *A*_control_ and *A*_sample_ are the absorbance of the control and sample, respectively. Inhibition concentration 50% (IC_50_) was also calculated.

### 2.7. Determination of Cytotoxic Properties Using Brine Shrimp Lethality Test Method

Cytotoxic activities to brine shrimp were determined following the methods of Itam et al. [9], Dastagir and Hussain [15] and Meyer et al. [16] with a few modifications. The shrimp larva was hatched using a small tank consisting of two compartments filled with sea water as the medium. *A. salina* shrimp eggs were added to the covered compartment, and a lamp was placed above the open side of the tank to attract hatched shrimps through perforations in the partition wall. Nauplii of the shrimps were ready to be used in cytotoxic assay after 48 hours of hatching. 10 mg of leaves and stem bark extracts were dissolved in methanol using a 10 mL volumetric flask to prepare a 1,000 *µ*g/mL stock solution. Various volumes of this stock solution were transferred using a micropipette into separate tubes to obtain extract solutions with various concentrations (31.25, 62.5, 125, 250, 500, and 1,000 *µ*g/mL). These solutions were left to dry at room temperature, and 50 *µ*L of DMSO and 2 mL of sea water were added to the residues to dissolve them. Ten nauplii were added into every mixture, and sea water was added to obtain 5 mL mixtures. 50 *µ*L of DMSO with sea water added till the volume of 5 mL was used as the control. The bioassay mixtures were left for 24 hours, after which the number of dead nauplii at every concentration was recorded and counted. The LC_50_ of extract was determined using probit value and regression equation.

### 2.8. Determination of Total Phenolic Content

Determination of the total phenolic content of hexane, ethyl acetate, and methanol *S. aqueum* leaves and stem bark extracts was done using the Folin–Ciocalteu method based on Sen et al. [13] and Itam et al. [9] with modifications. 10 mg of these extracts were dissolved in methanol to 10 mL volume in separate volumetric flasks. 0.5 mL of every extract solution was transferred into test tubes containing 1.0 mL of the Folin–Ciocalteu reagent using a micropipette. These mixtures were left for 5 minutes and were then added with 2.0 mL of 7% (w/v) sodium carbonate and water till the volume of these mixtures became 10 mL. The mixtures were whisked thoroughly. Each mixture was incubated for 2 hours at room temperature, and their absorbance was measured using the spectrometer UV/VIS 1700 Series at 760 nm. The calibration curve for the calculation of phenolic content in samples was plotted using gallic acid at various concentrations. Phenolic content was calculated as gallic acid equivalent per 10 mg of dried extracts.

## 3. Results and Discussion

### 3.1. Phytochemical Screening

**Figure 1:**
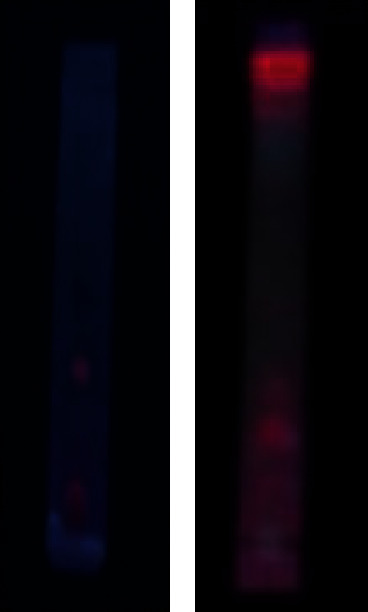
Results of thin-layer chromatography analysis in the identification of coumarins.

Phytochemical screening was performed to identify and ensure that these plants contain compounds that perform bioactivities, such as antioxidant and cytotoxic compounds. Phytochemical screening results on *S. aqueum* leaves and stem barks as presented in Table 1 show that the leaves of both variations of *S. aqueum* (with pink and red fruits) contained the same compounds, namely phenolics, flavonoids, and triterpenoids. As for *S. aqueum* stem barks, besides containing phenolics, flavonoids, and triterpenoids, the stem barks for both *S. aqueum* variations also contained saponins and alkaloids. This result suggested that the extracts of these plants showed antioxidant abilities. Phenolics and flavonoids donate hydrogen from their hydroxyl group to DPPH free radicals to inhibit the oxidation process [17]. Alkaloids also exhibit antioxidant properties as reported by Gutiérrez et al. [18], Gülçin et al. [19], and Benabdesselam et al. [20]. The result of thin-layer chromatography analysis in coumarins identification is shown in Figure 1. The absence of blue fluorescence indicated the absence of coumarins in samples.

### 3.2. Extraction of Leaves and Stem Bark of *S. aqueum*

To extract the leaves and stem bark of *S. aqueum*, the maceration method was used with methanol, ethyl acetate, and hexane solvents. As methanol, ethyl acetate, and hexane are solvents with different polarity, the chemical components that dissolve in these solvents will also be of different polarity. This results in the separation of polar, semipolar, and nonpolar compounds in samples based on solvent polarity. Results of these extractions are shown in Table 2, which expressed that the extract percentage followed the trend of methanol extracts > ethyl acetate extracts > hexane extracts, suggesting that this plant contained phenolics and/or flavonoids and alkaloids as their salt in the stem bark. As these are all polar compounds that dissolve in the polar methanol solvent, the percentage of methanol extract was the highest. This is compatible with the results of phytochemical screening in Table 1, which proved that leaves and stem barks contained flavonoids, phenolics, and alkaloids. By identifying the percentage of these extracts in the samples, the number of leaves or stem bark samples needed to obtain a certain amount of extracts could be calculated.

### 3.3. Determination of Antioxidant Activities Using DPPH Free Radical Method

**Figure 2:**
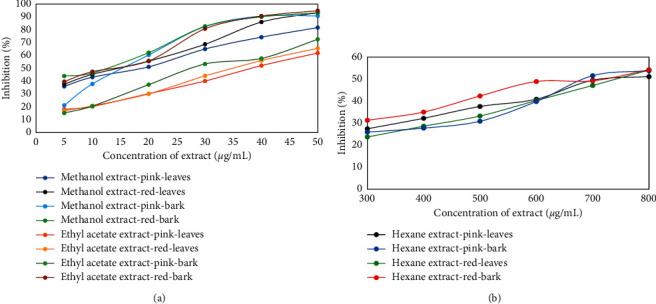
Antioxidant activities of various concentrations of *S. aqueum* leaves and stem bark extracts to DPPH radical: (a) methanol and ethyl acetate extracts and (b) hexane extracts.

The existence of a spare electron that causes delocalization in the entire molecule makes DPPH a stable free radical. The donation of antioxidant proton contained in the sample to DPPH reduced the violet appearance of DPPH due to the production of 1,1-diphenyl-2-picrylhydrazyn with a yellow appearance [21, 22]. The results showed that all extracts had antioxidant ability but in different inhibition percentages, as shown in Figure 2. Increasing extract concentration would cause the inhibition percentage of DPPH radical to also increase. The regression equation determined from these curves was used to calculate the IC_50_ value that represents the sample concentration needed to reduce DPPH by 50%. These IC_50_ values are presented in Table 3. The IC_50_ values in the order of hexane extracts > ethyl acetate extracts > methanol extracts, either in the leaves or stem bark of *S. aqueum*, suggested that the order of antioxidant activities progressed in the order of methanol extracts > ethyl acetate extracts > hexane extracts. However, based on the IC_50_ values for both the leaves and stem bark of *S. aqueum*, methanol and ethyl acetate extracts were both categorized as very powerful antioxidants, while hexane extracts showed no antioxidant activity. Antioxidant activity is considered very powerful if IC_50_ values are lower than 50 *μ*g/mL, strong if IC_50_ values fall between 50 and 100 *μ*g/mL, moderate if IC_50_ values fall between 101 and 250 *μ*g/mL, weak when IC_50_ values fall between 250 and 500 *μ*g/mL, and inactive if IC_50_ values are greater than 500 *μ*g/mL [23, 24]. As methanol and ethyl acetate are solvents that can dissolve polar organic components such as flavonoids or phenolics contained in the leaves and stem bark of *S. aqueum*, the concentrations of flavonoids and/or phenolics in these extracts were higher. Therefore, flavonoids and/or phenolics showed a positive correlation to antioxidant activities of extracts, as reported previously [25, 26]. Antioxidant activities of the leaves and stem bark extracts were lower than that of the reference compound of ascorbic acid, except for the stem bark extracts of red fruits.

Table 3 shows that the IC_50_ value of stem bark extracts were lower than that of leaves extracts, suggesting that antioxidant activities of stem bark extracts was stronger than that of leaves extracts, for methanol, ethyl acetate, and hexane extracts. This could be due to the presence of alkaloid compounds in stem bark extracts, which was proven via phytochemical screening (Table 1). Gutiérrez et al. [18], Gülçin et al. [19], and Benabdesselam et al. [20] also reported that alkaloids have antioxidant ability toward DPPH.

Previously, there is no study reported on the antioxidant activity of methanol, ethyl acetate, and hexane extracts of *S. aqueum* to DPPH, both leaves and stem barks, but the antioxidant activity of aqueous and ethanol extracts of *S. aqueum* leaves where IC_50_ values of aqueous and ethanol extracts were 0.33 and 0.21 mg/mL, respectively [3]. Therefore, aqueous extract is categorized as the weak antioxidant, while ethanol extract is categorized as the moderate antioxidant [24]. The present results suggested methanol and ethyl acetate extracts both were categorized as very powerful antioxidants. These categories are different because the solvent used for extraction is also different.

### 3.4. Determination of Antioxidant Activities Using Hydrogen Peroxide Method

**Figure 3:**
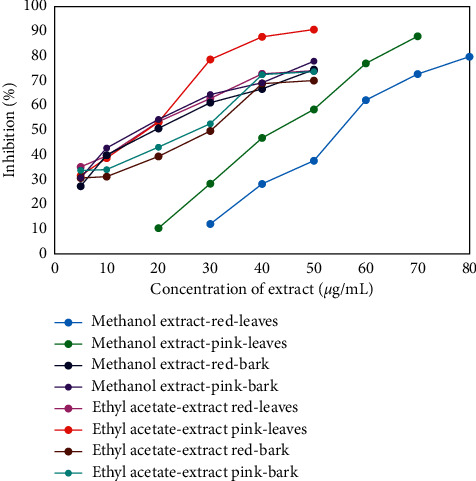
Antioxidant activities of various concentrations of *S. aqueum* leaves and stem bark extracts to hydrogen peroxide.

Results of antioxidant activities of leaves and stem bark extracts to hydrogen peroxide are shown in Figure 3, where all extracts exhibited antioxidant abilities. Increasing extract concentration of methanol and ethyl acetate resulted in increased antioxidant activities in either leaves or stem bark extracts. Hexane extracts on the other hand showed no reactivity. Their curves were not shown in the figure as hexane extracts only showed effects to hydrogen peroxide at concentrations greater than 1.000 *μ*g/mL. According to Jun et al. [23] and Mustarichie et al. [24], antioxidant activity is categorized as inactive when IC_50_ value is greater than 500 *μ*g/mL. IC_50_ values of all extracts are presented in Table 4. Based on these IC_50_ values, all extracts were very powerful antioxidants, except for the methanol extracts of leaves from plants with red fruits that fell under the strong category [23, 24]. IC_50_ of methanol extracts of stem bark was smaller than that of leaves, indicating that methanol extracts of stem bark showed stronger antioxidant activities than methanol extracts of leaves. This might be due to the presence of alkaloid in methanol extracts of stem bark as shown in Table 1. Besides exhibiting antioxidant activities to DPPH, alkaloids were also reported to portray antioxidant activities to hydrogen peroxide, as previously reported by Gülçin et al. [19]. Meanwhile, the IC_50_ of ethyl acetate extracts of stem bark was higher than that of leaves, indicating the stronger antioxidant activities of ethyl acetate extracts of leaves compared with stem bark. The total phenolic content of leaves extracts was also higher than that of stem bark extracts. Thus, the presence of alkaloid with antioxidant property contained in the stem bark could not be extracted by ethyl acetate solvent due to the more polar alkaloid.

### 3.5. Determination of Cytotoxic Properties Using Brine Shrimp Lethality Test Method

**Figure 4:**
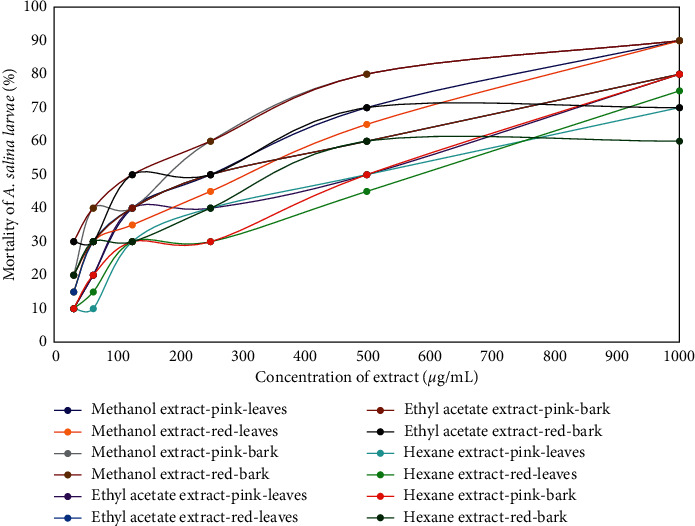
Graph of correlation between the various concentrations of leaves and stem bark extracts of *S. aqueum* and the mortality of *A. salina* larvae.

Cytotoxic properties of various *S. aqueum* extracts were evaluated using the larvae of *A. salina*. Results presented in Figure 4 showed that an increase in extract concentration would lead to increased mortality percentage of *A. salina* larvae. To express the level of sample cytotoxic property, the lethal concentration (LC_50_) value, defined as the concentration of compound or sample that can kill half of the sample population of a specific test animal, was used. A lower LC_50_ value shows higher cytotoxic activity of extracts. LC_50_ values of these extracts are presented in Table 5, which shows that all extracts were categorized with moderate cytotoxicity [27, 28]. This table also shows that the LC_50_ values of stem bark extracts were lower than those of leaves extracts, implying that the cytotoxicity of stem bark extracts was higher than that of leaves extracts, either in plants with pink or red fruits. Furthermore, among these extracts, methanol extracts showed the highest cytotoxic activities followed by ethyl acetate extracts and hexane extracts. Thus, stem bark methanol extracts of *S. aqueum* had the highest cytotoxic activities to *A. salina*.

### 3.6. Determination of Total Phenolic Content

**Figure 5:**
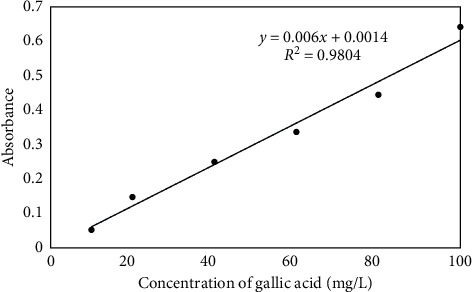
Calibration standard curve of gallic acid to determine total phenolic content.

The determination of total phenolic content used gallic acid as the standard compound, with the calibration curve shown in Figure 5. The curve shows that increasing gallic acid concentration also increases absorbance, and their correlation is linear with the regression equation *y* = 0.006*x* + 0.0014 (*R*^2^ = 0.9804). The total phenolic contents in leaves and stem bark extracts of *S. aqueum* calculated using this gallic acid standard calibration curve are shown in Table 6. The table shows that the total phenolic content of leaves extracts was higher than that of stem bark extracts, for methanol, ethyl acetate, and hexane extracts. The total phenolic content was in the order of methanol extracts > ethyl acetate extracts > hexane extracts for both leaves extracts and stem bark extracts.

Methanol extracts of both leaves and stem bark showed the highest bioactivities (cytotoxic properties to *A. salina* larvae, and antioxidant activities to DPPH and hydrogen peroxide) followed by ethyl acetate extracts and hexane extracts. Methanol extracts also contained the highest total phenolics followed by ethyl acetate and hexane extracts, indicating that an increase in phenolic content of the extracts would increase the antioxidant activity.

Phenolics are potent antioxidants to DPPH due to the donation of hydrogen of the phenolic hydroxyl group. It has been previously reported that there is a positive correlation between antioxidant activity to DPPH and phenolic content of extract, where an increase in total phenolic content increases free radical scavenging activity. Sen et al. [13] reported a correlation between the phenolic content in methanol extracts of *Meyna spinosa* Roxb. leaves and antioxidant activity, where methanol extracts containing the highest phenolics exhibit the highest antioxidant activities. Nyein et al. [29] reported that the high phenolic content of *Terminalia chebula* flowers shows a linear correlation to antioxidant activity where the IC_50_ value of *T. chebula* flowers is lower. They also reported higher phenolic content in *T. chebula* fruits extracts with lower IC_50_ values compared with *T. chebula* leaves extracts, indicating that phenolic content and antioxidant activity have a positive correlation.

Previously, there is no study reported on the total phenolic content of methanol, ethyl acetate, and hexane extracts of *S. aqueum*, both leaves and stem barks, but on the total phenolic content of aqueous and ethanol extracts of *S. aqueum* leaves that were 180 and 520 mg/g, respectively [3]. These results are different from present results because the solvent used for extraction is also different.

## 4. Conclusion

Leaves of *S. aqueum* with either pink or red fruits contained secondary metabolites, namely flavonoids, phenolics, and triterpenoids, while their stem barks contained not only flavonoids, phenolics, and triterpenoids but also saponins and alkaloids. Leaves and stem bark extracts of these plants with either pink or red fruits exhibited antioxidant activities to DPPH and hydrogen peroxide and cytotoxic activities to *A. salina*. Methanol and ethyl acetate extracts were categorized as very powerful antioxidants to DPPH and hydrogen peroxide, while hexane extracts did not show antioxidant activities. Cytotoxic activities of these extracts to *A. salina* were categorized as moderate. Total phenolic contents of these extracts were in the order of methanol extracts > ethyl acetate extracts > hexane extracts for both leaves and stem bark extracts. In this case, the total phenolic content of leaves extracts was higher than that of stem bark extracts. In general, the stem bark extracts of *S. aqueum* possessed higher antioxidant and cytotoxic activities than the leaves extracts of *S. aqueum*.

## Figures and Tables

**Table 1:** Results of phytochemical screening of leaves and stem bark extracts of *S*. *aqueum* with pink and red fruits.

No.	Phytochemicals	Fruit color of leaves extract		Fruit color of bark extract	
		Pink	Red	Pink	Red
(1)	Phenolics	+	+	+	+
(2)	Flavonoids	+	+	+	+
(3)	Saponins	−	−	+	+
(4)	Steroids	−	−	−	−
(5)	Triterpenoids	+	+	+	+
(6)	Coumarins	−	−	−	−
(7)	Alkaloids	−	−	+	+

The presence of compounds was denoted by +, meanwhile the absence of compounds was denoted by −.

**Table 2:** Maceration results of *S. aqueum* leaves and stem barks using different solvents.

No.	Solvent	Percentage of extract (%)			
		Fruit color of leaves extract		Fruit color of bark extract	
		Pink	Red	Pink	Red
1.	Methanol	22.87	16.24	7.58	6.48
2.	Ethyl acetate	4.32	4.36	2.90	2.44
3.	Hexane	1.75	1.14	0.52	0.54

**Table 3:** Antioxidant activities 50 (IC_50_) of *S. aqueum* leaves and stem bark extracts to DPPH free radicals.

No.	Extract	IC_50_ (*μ*g/mL) to DPPH			
		Fruit color of leaves extract		Fruit color of bark extract	
		Pink	Red	Pink	Red
(1)	Methanol	17.59	14.47	17.14	9.71
(2)	Ethyl acetate	38.69	35.72	31.52	12.09
(3)	Hexane	756.45	748.30	736.78	689.23
(4)	Ascorbic acid (positive control)	9.75			

**Table 4:** Antioxidant activities 50 (IC_50_) of *S. aqueum* leaves and stem bark extracts to hydrogen peroxide.

No.	Extract	IC_50_ (*μ*g/mL) to H_2_O_2_			
		Fruit color of leaves extract		Fruit color of bark extract	
		Pink	Red	Pink	Red
(1)	Methanol	44.02	55.85	19.13	22.37
(2)	Ethyl acetate	16.44	18.99	24.21	27.50
(3)	Hexane	No reactive	No reactive	No reactive	No reactive

**Table 5:** Lethal concentration 50 (LC_50_) of leaves and stem bark extracts of *S. aqueum* to *A. salina* larvae.

No.	Extract	LC_50_ (*μ*g/mL) to BSLT			
		Fruit color of leaves extract		Fruit color of bark extract	
		Pink	Red	Pink	Red
(1)	Methanol	202.22	198.06	134.52	104.04
(2)	Ethyl acetate	286.42	225.52	192.26	174.47
(3)	Hexane	420.42	440.65	356.53	396.93

**Table 6:** Total phenolic content (TPC) of leaves and stem bark extracts of *S. aqueum* with pink and red fruits.

No.	Extract	TPC (mg GAE/10 mg dried extract)			
		Fruit color of leaves extract		Fruit color of bark extract	
		Pink	Red	Pink	Red
(1)	Methanol	10.61	9.56	3.35	3.38
(2)	Ethyl acetate	8.73	7.91	2.63	3.01
(3)	Hexane	3.51	3.98	1.68	2.14

## Data Availability

The data used to support the findings of this study are available upon request to the authors.
